# A 10-year monitoring of soil properties dynamics and soil fertility evaluation in Chinese hickory plantation regions of southeastern China

**DOI:** 10.1038/s41598-021-02947-z

**Published:** 2021-12-07

**Authors:** Jin Jin, Luoqi Wang, Karin Müller, Jiasen Wu, Hailong Wang, Keli Zhao, Frank Berninger, Weijun Fu

**Affiliations:** 1 State Key Laboratory of Subtropical Silviculture, Zhejiang A&F University, Hangzhou, 311300 China; 2 The New Zealand Institute for Plant and Food Research Limited, Ruakura Research Centre, Private Bag, Hamilton, 3123 New Zealand; 3 Biochar Engineering Technology Research Center of Guangdong Province, School of Environmental and Chemical Engineering, Foshan University, Foshan, 528000 China; 4 Department of Environmental and Biological Sciences, University of Eastern Finland, PO Box 111, 80101 Joensuu, Finland; 5 Zhejiang Provincial Key Laboratory of Carbon Cycling in Forest Ecosystems and Carbon Sequestration, Zhejiang A&F University, Lin’an, 311300 China

**Keywords:** Biogeochemistry, Environmental sciences

## Abstract

Monitoring the temporal and spatial variation of soil properties is helpful to understand the evolution of soil properties and adjust the management method in time. Soil fertility evaluation is an urgent need to understand soil fertility level and prevent soil degradation. Here, we conducted an intensive field investigation in Chinese hickory (*Carya cathayensis* Sarg.) plantation to clarify the spatial and temporal variation of soil properties and its influencing factors, and to evaluate the change of soil fertility. The results showed that the soil pH and soil organic carbon (SOC) significantly increased from 2008 to 2018, while available nitrogen (AN) significantly decreased from 2008 to 2018. The semi-variance revealed that except available phosphorus (AP), the spatial dependencies of soil properties increased from 2008 to 2018. An increasing south-north gradient was found for soil AN, AP, available potassium (AK) and SOC and a decreasing south-north gradient was found for soil pH. The average soil fertility in the whole area was increased from 2008 to 2018. Our findings demonstrated that the changes of the management measures were the reason for the change of soil properties from 2008 to 2018. Therefore, rational fertilization strategies and sod cultivation are recommended to maintain the long-term development of the producing forest.

## Introduction

Soil is the foundation of life and biodiversity in the ecosystem, and plays an important role in nutrient cycling. Proper soil management can improve food quality and safety, maintain or improve soil fertility levels and avoid soil degradation^1,2^. From this perspective, long-term monitoring of changes in soil physiochemical properties contributes to make informed decisions for sustainable land use. However, extensive characterization of soil properties are difficult, as many studies demonstrated that soil properties were spatially and temporally different^3–5^. The spatial variation of soil properties in ecosystem are mainly related with the combined effects of geological, climatic, human activities and so on^6–10^. Globally, soil organic carbon (SOC) and total N are positively correlated with precipitation and negatively correlated with temperature^11,12^. Soils with a high proportion of clay produce more biomass and protect more organic matter through the association of soil aggregates and organic minerals, resulting in an increase in SOC content in the soil^8^. However, the influence of natural factors on soil properties are relatively slow, but human activities have dramatically altered the rate of supply of many major nutrients and have thus led to rapid soil property changes^13,14^.

China, the largest developing country in the world, has experienced a rapid shift from natural forests into production forests^15^. Since the 1960s, forest plantations have been expanding in order to meet the population's growing demand for forest products and ecological services. By 2000, the total area of forest plantation was 20.57 million ha, which contributed about 90% of the forest area expansion and 49.3% of forest carbon sink^16,17^. Thus, production forests are critical to promoting economic development and participating in C balance at regional and national scales^18^. Intensive management (including chemical fertilization, undergrowth removal, and herbicide application) is a way of managing production forests to drive growth in plantation productivity and economic returns^19^. Unfortunately, long term intensive management have led to soil degradation, reduced microbial diversity and soil nutrients imbalances^20–23^. Previous study showed that human activities have significantly changed the spatial pattern of soil nutrients in *Torreya grandis* plantation, resulting in uneven spatial distribution of soil fertility^24^. Under the long-term intensive management, the soil texture of moso bamboo forest had clear spatial heterogeneity, which affected the productivity of moso bamboo forest^25^. Spatial variation of soil properties also appeared in the soil of *Malus pumila* (apple), *Camellia sinensis* (tea), *Hevea brasiliensis* (rubber), *Eucalyptus robusta* (eucalyptus) and *Castanea mollissima* (Chinese chestnut) plantations^26–30^. Therefore, it is necessary to understand the spatial variation of soil properties and identify areas with low or high soil fertility, which could further guide site-specific land management strategies.

Geostatistical studies have proved to be useful tools for analyzing spatial and temporal variability of soil physicochemical properties^31,32^. Because of its ability to quantify and reduce sampling uncertainty and minimize survey costs. As the important indicators of soil fertility, the spatial distribution of soil pH, soil available nitrogen (AN), soil available phosphorus (AP), soil available potassium (AK) and soil SOC has been widely studied. In this regard, Liu et al.^33^ identified the determinants of SOM distribution in an intensive agricultural region of northeastern China by kriging methods. Liu et al. (2009) studied the spatial distribution characteristics of soil properties, and guided the fertilization and management at specific sites^34^. In addition, geostatistics can also be used to investigate the spatial patterns of soil properties’ change over time^35^. These published papers have analyzed the heterogeneity of soil properties in farmland, cultivated land and Loess Plateau region^36–39^, while less attention is focused on soil properties in plantation forests of Southeastern China.

Chinese hickory (*Carya cathayensis* Sarg.) is a unique edible nut and woody oil species that distributed in the Tianmu mountain in Zhejiang province of southeastern China. Because of its unique taste and high nutritional value, the area of Chinese hickory has reached 93,300 ha with a total yield of 31,500 t in 2018^40,41^. For the purpose of improving the yield of Chinese hickory, farmers have adopted intensive agricultural management with extensive application of chemical fertilizer^42–46^. In addition, it was necessary to remove undergrowth (herbicide or artificial weeding) in order to harvest Chinese hickory fruits (Fig. S1). Therefore, it is necessary to better understand the spatio-temporal variation of soil properties in Chinese hickory plantation regions in order to guide sustainable Chinese hickory management. We hypothesized that under the influence of different factors, soil fertility would change progressively with the increase of intensive management years. The objectives of our study were to (1) investigate the spatio-temporal variation of soil properties; (2) to explore the causes of soil properties changes; (3) to evaluate the change of soil fertility in Chinese hickory plantation regions.

## Results

### Descriptive statistics

**Figure 1:**
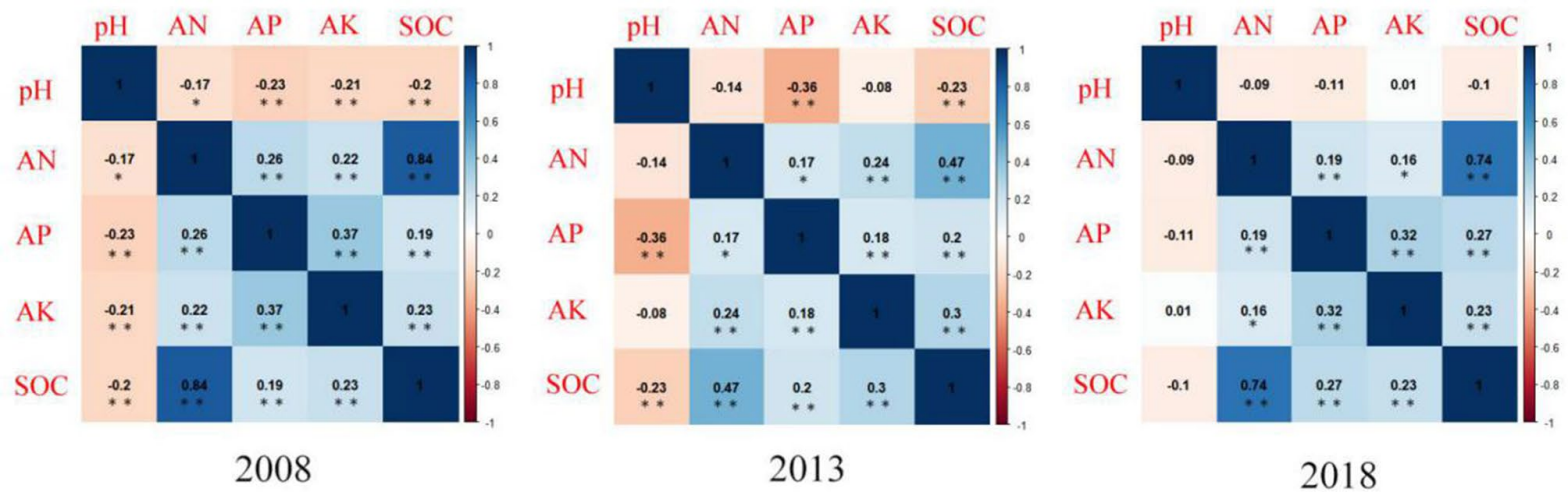
Pearson correlation among soil properties in 2008, 2013 and 2018. Color depicts the direction of the correlation (blue = positive, red = negative). P-values in black color are significant (*P < 0.05, **P < 0.01). The correlation coefficients are shown in the panel. *AN* available nitrogen, *AP* available phosphorus, *AK* available potassium, *SOC* soil organic carbon.

After Box-cox transformation, the soil properties all passed the K-S test (*K-S*_*P*_ > 0.05) (Table 1). The coefficient of variation (CV) values ranged from 10 to 130%. According to Fu et al. (2014) reported^47^, CV < 10%, between 10 and 90%, and > 90% indicate low, moderate and high variabilities, respectively. With the exception of AP concentrations for 2008 and 2018, which were highly variable, all other soil properties were moderately variable. The average pH was significantly higher in 2018 than that in 2008 (*P* < 0.05). From 2008 to 2018, the AN concentration declined by 38% (Table 1). The concentration of AK in 2018 was significantly higher than in 2013 (Fig. S2). The variation ranged of AN and AP concentrations were significantly narrower in 2018 than in 2008 (Table 1). The soil pH was negatively correlated with AP and SOC concentrations in 2008 and 2013 (*P* < 0.01, Fig. 1). Correlations between AN, AP, AK and SOC in 2008–2018 were positive (Fig. 1).

**Table 1:** Descriptive statistics of the soil attributes.

Attributes	Year	Minimum	Maximum	Range	CV%	Mean ± SD	Skewness	Kurtosis	*K-S*_*p*_
pH	2008	4.09	7.58	3.49	13.07	5.51 ± 0.72b	0.24	− 0.24	0.01 (0.20)
	2013	4.26	7.12	2.86	10.92	5.31 ± 0.58c	0.04	− 0.23	< 0.01 (0.20)
	2018	4.50	7.35	2.85	10.23	5.77 ± 0.59a	0.02	− 0.14	< 0.01 (0.20)
AN (mg kg^−1^)	2008	83.69	375.10	291.41	31.97	190.08 ± 60.76a	− 0.02	− 0.35	0.03 (0.20)
	2013	36.21	348.30	312.09	32.21	171.12 ± 55.12b	0.01	− 0.13	< 0.01 (0.20)
	2018	24.50	147.31	122.81	32.54	71.89 ± 23.39c	− 0.12	0.15	< 0.01 (0.20)
AP (mg kg^−1^)	2008	0.04	21.20	21.16	129.60	3.75 ± 4.86b	0.02	− 0.41	< 0.01 (0.20)
	2013	0.10	15.84	15.74	82.50	4.40 ± 3.63a	− 0.69	1.12	< 0.01 (0.20)
	2018	0.06	15.44	15.38	91.25	4.00 ± 3.65ab	0.06	− 0.50	< 0.01 (0.20)
AK (mg kg^−1^)	2008	31.21	246.09	214.88	46.86	101.37 ± 47.50a	0.06	− 0.50	< 0.01 (0.20)
	2013	5.96	140.08	134.12	54.79	50.98 ± 27.93b	0.16	− 0.31	< 0.010 (0.20)
	2018	22.06	340.39	318.33	60.78	114.60 ± 69.65a	− 0.05	− 0.75	< 0.01 (0.10)
SOC (g kg^−1^)	2008	3.06	42.42	39.36	40.28	18.32 ± 7.38b	− 0.40	0.69	< 0.01 (0.20)
	2013	6.06	38.66	32.60	34.62	18.34 ± 6.35b	− 0.15	− 0.05	< 0.01 (0.20)
	2018	3.14	46.96	43.82	38.92	21.30 ± 8.29a	− 0.74	1.29	0.03 (0.08)

*AN* available nitrogen, *AP* available phosphorus, *AK* available potassium, *SOC* soil organic carbon.

Different letters in the same variable indicate significant differences among years at *P* < 0.05 level. CV: coefficient of variation; *K-S*_*p*_: significance level of Kolmogorov–Smirnov test for normality. The *K-Sp* values in brackets were calculated after transformation.

### Spatial cluster and spatial outlier analysis

**Figure 2:**
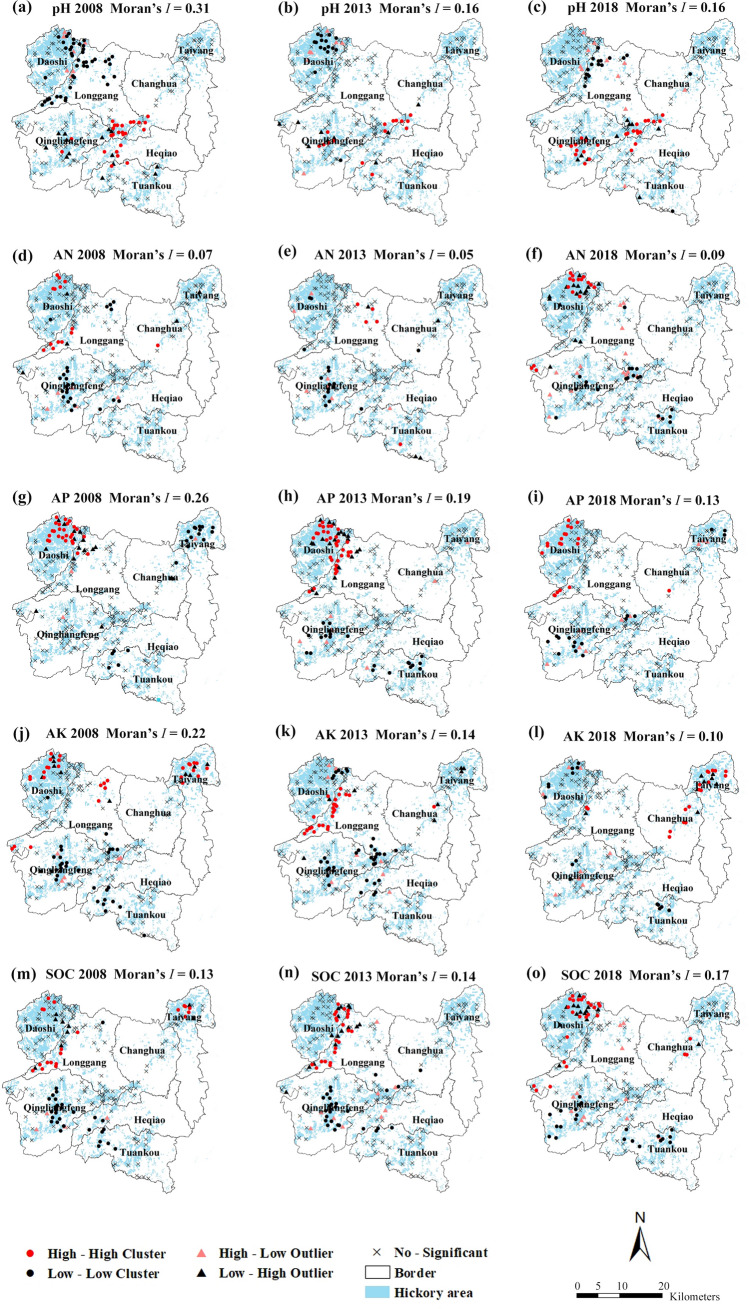
Local indicators of spatial association (LISA) maps of soil properties in Chinese hickory plantation regions. *AN* available nitrogen, *AP* available phosphorus, *AK* available potassium, *SOC* soil organic carbon. The maps were created in ArcGIS desktop 10.7.

The local indicators of spatial association (LISA) maps (Fig. 2) indicated a significant positive spatial autocorrelations for all soil properties (*P* < 0.05). The local Moran's *I* result identified high-high spatial clusters of soil pH in the middle region of the study area, while low-low clusters of soil pH were distributed in the northwest region of the study area (Daoshi town) from 2008 to 2018 (Fig. 2a–c). On the contrary, high-high clusters of AN, AP and SOC were mainly located in the northwest region of study area (Fig. 2d–i, m–o). Meanwhile, high-high clusters of AK concentration shifted from northwest to northeast of the study area from 2013 to 2018 (Fig. 2k–l).

To further describe the spatial structures of soil properties in 2008, 2013 and 2018, we calculated the semi-variances function of each study variable and selected the best-fitted models and their related parameters (Table 2). The spatial dependencies (C_0_/C_0_ + C) for soil pH, AN, AK and SOC were moderate and strong for AK in 2008 and 2013. The spatial autocorrelation for SOC was improved from 2008 to 2018 (Table 2). The ranged of soil properties varied from 0.16 km (AP) to 40.7 km (pH) in 2008, and from 0.13 km (AN) to 23.73 km (AP) in 2018, respectively.

**Table 2:** Theoretical semi-variance models and their corresponding parameters of soil properties in 2008, 2013 and 2018.

Attributes	Theoretical model	Year	Nugget (C_0_)	Sill (C_0_ + C)	Nugget/sill	Range (km)	R^2^
pH	Exponential	2008	0.36	0.71	0.51	40.70	0.91
	Exponential	2013	0.16	0.32	0.50	3.39	0.85
	Gaussian	2018	0.07	0.35	0.20	0.19	0.75
AN (mg kg^−1^)	Exponential	2008	1606.00	3642.00	0.44	2.16	0.76
	Exponential	2013	2845.30	3393.14	0.84	4.82	0.73
	Exponential	2018	111.00	633.80	0.18	0.13	0.50
AP (mg kg^−1^)	Exponential	2008	2.70	25.74	0.10	0.16	0.87
	Gaussian	2013	1.92	12.79	0.15	1.61	0.64
	Linear	2018	1.54	1.88	0.82	23.73	0.80
AK (mg kg^−1^)	Exponential	2008	1972.40	2784.92	0.71	0.85	0.85
	Exponential	2013	517.67	737.80	0.70	5.18	0.78
	Exponential	2018	1490.00	4981.00	0.30	3.78	0.81
SOC (g kg^−1^)	Gaussian	2008	42.60	85.21	0.50	17.42	0.88
	Gaussian	2013	34.00	68.01	0.50	23.21	0.84
	Gaussian	2018	0.10	65.99	0.01	5.40	0.91

*AN* available nitrogen, *AP* available phosphorus, *AK* available potassium, *SOC* soil organic carbon.

**Figure 3:**
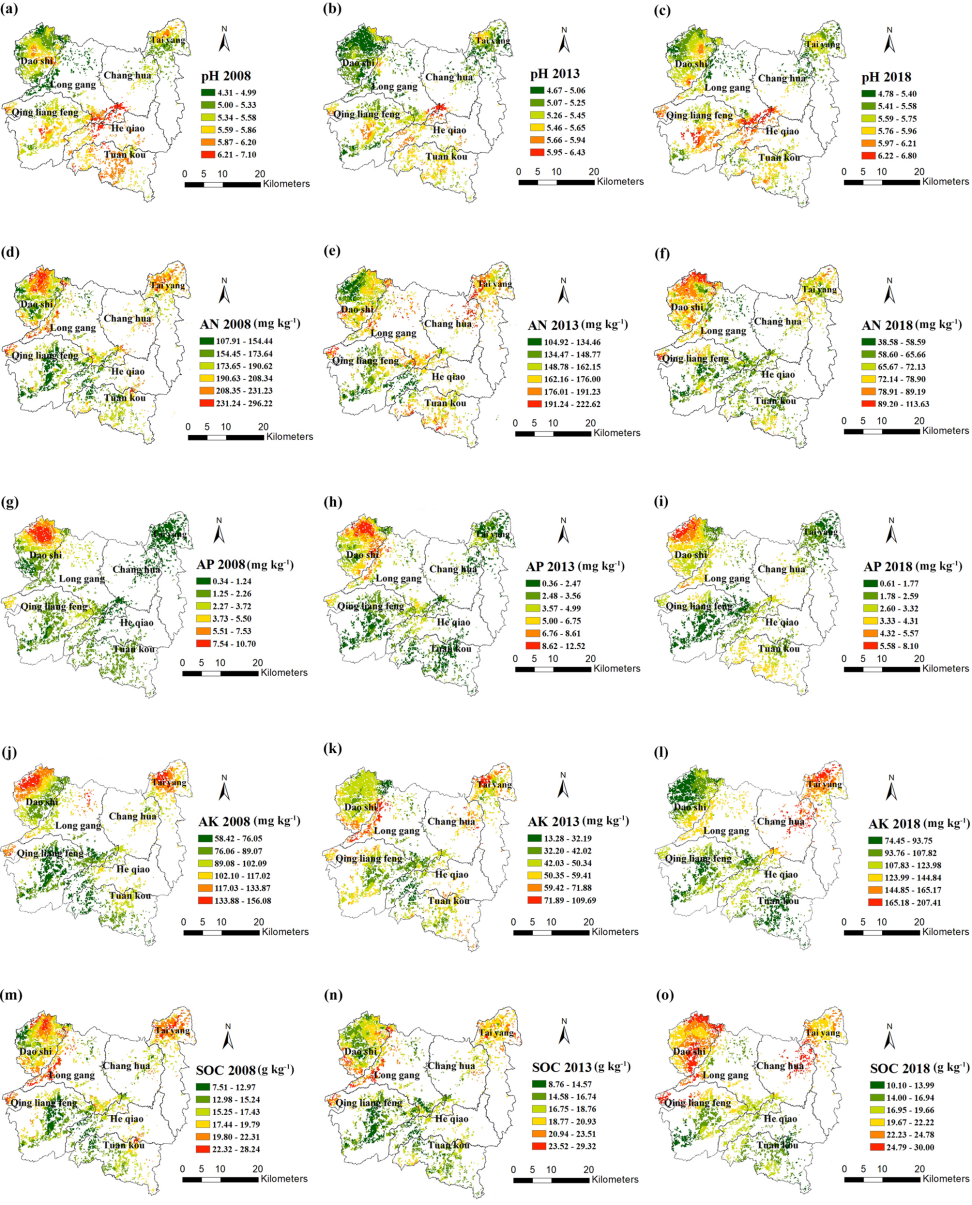
Spatial distribution maps of soil properties in Chinese hickory plantation regions. *AN* available nitrogen, *AP* available phosphorus, *AK* available potassium, *SOC* soil organic carbon. The maps were created in ArcGIS desktop 10.7.

The spatio-temporal distribution maps of soil properties were revealed by the ordinary Kriging interpolation method based on the semi-variance models for 2008, 2013 and 2018. The concentrations of AN, AP, AK and SOC had similar spatial distribution patterns (Fig. 3d–o), with high values mainly located in the northwest and northeast parts of the study area, while low values in the central and south regions. However, pH values showed an opposite spatial distribution pattern with a gradually increasing trend from north to south (Fig. 3a–c). Generally speaking, the spatial distributions of soil properties were similar to the above spatial clusters identified by local Moran’s *I* (Fig. 2). Meanwhile, soil properties varied considerably from 2008 to 2018 (Fig. S3). The pH value generally increased, with the largest increase in the western part of the study area in 2008–2018 (Fig. S3a–c). Contrary to soil pH, soil AN concentration decreased in study regions, and the dropped trend declining from 40 to 200 mg kg^−1^ (Fig. S3d–f).

### Control factors for soil properties

One way ANOVA analysis indicated that MAP and MAT had a significant influence on the change of pH (Table S2). What’s more, altitude has a significant influence on AK and SOC. However, most of soil properties was significantly influenced by anthropogenic factors such as fertilization, weeding, and harvesting methods (Table S2). This showed that the difference of comprehensive management mode, such as management method and intensity, will lead to the change of soil properties.

### Comprehensive appraisement of soil fertility

**Figure 4:**
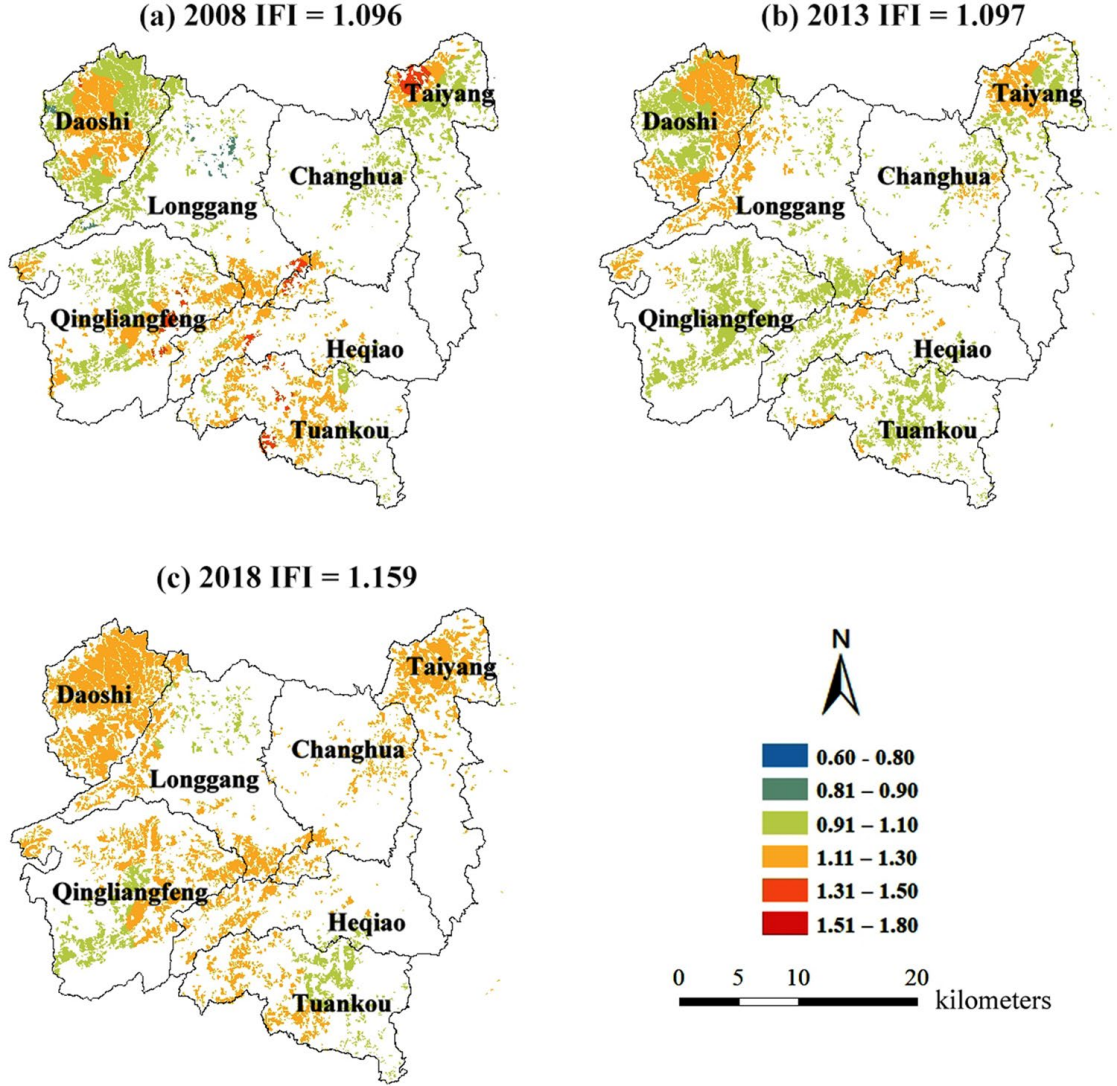
Soil integrated fertility index (IFI) level distribution map of the Chinese hickory plantation regions. The maps were created in ArcGIS desktop 10.7.

The improved Nemerow method was used to evaluate the soil integrated fertility index (IFI) of Chinese hickory plantation, and the results were shown in Fig. 4. The soil fertility of Chinese hickory plantation was at medium level, but the IFI value increased year by year, which was IFI = 1.096 in 2008, IFI = 1.097 in 2013 and IFI = 1.156 in 2018. In 2008, areas with IFI < 0.9 accounted for 21.5% of the whole study area. With the increase of years, areas with IFI < 0.9 gradually decreased, accounting for 11.5 and 8.6% of the whole study area in 2013 and 2018, respectively (Fig. 4). Compared with the previous two periods, IFI in most areas of the study area was between 1.1 and 1.3 in 2018. These results indicated that the soil fertility in the study area increased gradually with the increase of years, and the heterogeneity of soil fertility in different areas decreased (Fig. 4).

## Discussion

In this study, we monitored the temporal and spatial variability of soil properties and soil fertility in hickory plantations under intensive management for 10 years. Geostatistical studies have determined the degree of variability of soil properties in different regions and the degree of spatial dependence of soil properties. Kriging interpolation and exploratory spatial analysis can provide data support for regional soil management policies.

Soil pH is a fundamental property that has significantly influences on numerous soil physical, chemical, biological properties and processes. Therefore, it is considered to be a key soil variable in terrestrial ecosystem^48,49^. Long-term monitoring showed that the soil pH decreased significantly from 2008 to 2013 (Table 1). Previous study showed that long-term agricultural activities lead to the decreased of soil pH^50^. It is well known that excessive application of inorganic fertilizer is an important cause of soil acidification^51,52^. NH_4_^+^ ions in the fertilizer replace the basic cations (Ca^2+^, Mg^2+^, K^+^, Na^+^) bound on the soil surface, making it easier to leach from the soil and reducing its buffering effect on acidification. In addition, when an NH_4_^+^ ion is absorbed by the crop, an H^+^ ion is released into the soil solution, causing soil acidification. More serious, Al^3+^ increases in concentration in more acidic soils^53^. For hickory, soil acidification increased the risk of canker diseased, leading to mass mortality of hickory plantations^54^. This may be the reason that the decrease of soil pH in 2013.The global problem was recognized by local governments, which instructed farmers drastically reduced the amount of inorganic fertilizer applied, and added soil conditioners (lime, bamboo char and potassium humate) to raise the pH of the hickory soil in 2010^55^. Our study also found that precipitation had a significant effect on soil pH (Table S2). The reason is that precipitation can accelerate the decomposition of litter and promote the return of more cations to the topsoil to supplement the soil exchangeable cationic pool, thereby increasing the soil pH^56,57^. This may be the reason that the increase of soil pH in 2018. Kriging interpolation showed that soil pH value in the northwest part of the study area increased most during this decade. (Fig. S3a–c). This may be due to the steep slope and thin soil layer thickness of hickory plantations in the northwest of the study area, and the rapid continuous and rapid weathering of parent material supplementing rich base cation exchange, thus improving the soil buffer capacity^52^.

Descriptive analysis showed that soil AN decreased significantly from 190 to 71 mg kg^−1^ in this decades. According to the classification levels of the State Soil Survey Service of China (SSSSC 1996)^58^, the concentration of AN more than 120 mg kg^−1^ can be considered as high level. At the same time, Kriging interpolation analysis showed that high levels of AN were observed in almost the whole study area in 2008 and 2013 (Fig. 3). Lu^59^ concluded that soil AN in southern China is not a limiting nutrient, because the overuse of nitrogen fertilizer leads to the excess of soil AN. Geisseler and Scow's survey of 10 years long-term monitoring sites in China showed that soil pH was significantly reduced by 0.45–2.20 units during 8–25 years when inorganic N, P and K were applied^60^. This was consistent with the negative correlation between soil pH and AN, AP and AK. Zhao et al.^61^ found that when the amount of nitrogen applied was greater than the amount required by plants, further nitrogen application would be unfavorable to plant growth and might even limit the uptake of nitrogen by plants. Lei et al.^62^ showed that excessive accumulation of nitrogen in the soil would cause an increase in the incidence of canker diseased in Chinese hickory plants, which could lead to plant death (Fig. S4). In addition, in southeast of China, where the temperature is high and the rain is heavy, excessive nitrogen input significantly increased the leaching of reactive nitrogen and gas emissions, causing environmental pollution. Based on this, farmers reduced the amount of fertilizer they applied, and soil nutrients were taken away with the hickory harvest^57^. At the same time, the soil erosion of hickory plantations on the steep slope and exposed surface also caused the loss of nutrients. This significantly reduced the content of available nutrients, especially AN in 2018.

Compared with pH, AN, AP and SOC, the concentration of AP showed a higher variability (90%-120%). According to LISA map, the distribution of AP presented a serious polarization phenomenon, while the high-high clusters were mainly distributed in the northeastern and the low-low clusters were mainly distributed in the southeastern of study area (Fig. 2g–i). This was related to the relatively low phosphorus concentration derived from the parent materials. In addition, phosphorus was adsorbed and fixed by soil clay or calcium carbonate, so phosphate was less lost in soil runoff. However, in low soil pH, the active iron and aluminum in the soil increased, and the precipitation of iron and aluminum phosphate was easy to immobilized, resulting in the decrease of the availability of phosphorus^63^. Bruun et al.^64^ suggested that additional P fertilizer was not needed because P fertilizer has a strong aftereffect and higher temperature will increase soil phosphorus availability when soil available phosphorus was higher than 10.0 mg kg^−1^. Unfortunately, the AP concentration in the most of study area was less than 10 mg kg^−1^ from 2008 to 2018 indicating a severe phosphorus deficiency (Fig. 3g–i). Therefore, it is necessary to continue to apply phosphate fertilizer in regions with low P to improve or at least maintain hickory yields because of its poor mobility in soil^65^. Tong et al.^66^ reported that when AK was higher than 100 mg kg^−1^ in soil, it could meet the requirements of carbohydrate transportation and fat synthesis during the growth period of Chinese hickory nuts. However, the AK < 100 mg kg^−1^ were widely found in Daoshi town, Qingliangfeng town and Tuankou town (Fig. 3l). The soil in these areas were still potassium-deficient because potassium fertilizer supply was still below consumption. More specifically, Chinese hickory growing areas in these towns shared a common characteristic: it used to grow on steep slopes greater than 40 degrees and had serious soil erosion (field survey data). This will cause potassium loss with water runoff or leaching to deep layers. Therefore, increasing the application of potassium fertilizer, improving the availability of phosphorus and adjusting the fertilization structure are the key points for the sustainable development of hickory plantations.

SOC is commonly considered as an important indicator for evaluating soil fertility^67–70^. It can not only maintain and release soil nutrients, but also improve the physical structure of soil^71–73^. Table 2 showed that compared with 2008 and 2013, low "Nugget-to-sill" ratio was found in 2018 because the intrinsic soil management factors probably enhanced the spatial correlation due to the weakening of farmer’s intensive management after 2010. This was consistent with Chen et al. (2019) that long-term large-scale planting can reduce the spatial variability of soil properties and homogenize soil properties^37^. In our study, the concentration of SOC has significantly increased from 18.5 to 21.4 g kg^−1^ from 2008 to 2018 (Fig. S2). Moreover, the increase of soil SOC was the largest in Daoshi town and Qingliangfeng Town. The average elevation of these towns is over 500, and some are as high as 1000 m. Bangroo et al. (2017)^74^ reported that with the increase of altitude, the decrease of microbial activity resulted in the increase of SOC stability, which was one reason for the increased of SOC. The decrease of annual application of inorganic fertilizers under intensive management reduced the activity of microorganisms and the mineralization of SOC which resulted in the increased of SOC content. The traditional method of collecting Chinese hickory fruit required that the ground be kept clean, so herbicides have been the first choice for farmers to clear undergrowth in the past (Fig S1a,b). However, in recent years, due to the intensification of soil erosion, farmers have gradually changed their harvesting methods from knocking to laying nets (Fig. S1a–d). This method did not require the removal of undergrowth and thus reduced the application of herbicides. This enhanced the stability of soil aggregates^28,75^. Moreover, according to field surveys, white clover, ryegrass and milk vetch were grown in large quantities in these areas. The undergrowth’s fine root, root hair, associated mycorrhizal and root secretion of C will directly into microaggregate, provide physical protection against SOC mineralization and effectively increase soil C content^76^. Qian et al.^77^ study confirmed that soil organic carbon content in hickory plantations increased by 12.9% after 2 years of planting grass. There was a negative correlation between soil pH and SOC (Fig. 1), which was due to the reduced activity of fungi at lower soil pH, thus reducing the decomposition of organic matter^78^. Table 2 showed that forest age had a significant effect on soil pH and SOC. The reason may be that with the increased of forest age, soil surface litter increased and microorganisms promoted the decomposition of litters, thus the SOC content increase. However, the decomposition of litter would produce acid, which has a negative impact on soil pH. SOC is an important indicator of soil fertility, its depletion can trigger or increase soil degradation processes. Therefore, it is necessary to adopt sod cultivation, increase the application of organic fertilizer and carry out continuous monitoring in areas with low soil organic matter content.

Previous study showed that soil fertility varied significantly under different cropping systems^37^. And there was a significant correlation between soil fertility and rice crop yield, suggesting that soil health was crucial for sustainable crop production^65^. In our study, the overall soil fertility of study area belonged to the middle level in 2008–2018. Though soil fertility increased slightly in 2018 due to changes in land management intensity since 2010 (Fig. 4). However, the uneven spatial distribution of soil properties exists (Fig. 3). This required variable fertilization rates to maintain soil nitrogen, phosphorus, potassium and organic carbon balance. Soil AP and AK can be increased by adding additional potassium fertilizer and phosphate fertilizer. Soil conditioner (biochar, potassium humate) are also effective in reducing soil acidification. Sod cultivation will be an effective means to increase SOC. At present, production forests (Chinese Torreya and bamboo) are being intensively managed, including heavy application of chemical fertilizers and herbicides such as glyphosate^19,21^. Such intensive management measures seriously harm the ecological balance and further threaten the health of plants. Therefore, rational fertilization strategies and sod cultivation are recommended to maintain the long-term development of the producing forest.

## Materials and methods

### Study area

**Figure 5:**
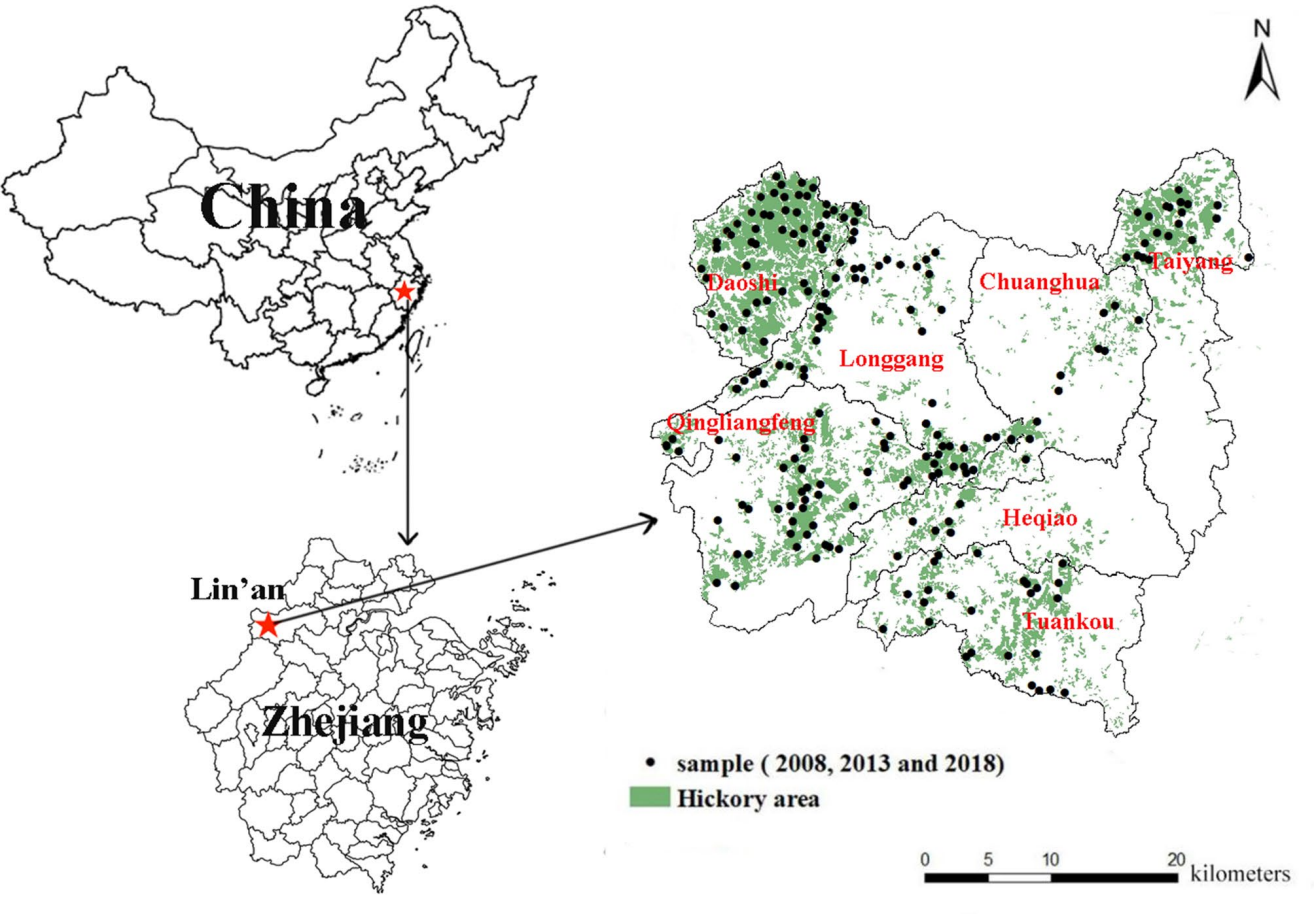
Location of the study area in Lin’an city, Zhejiang Province, China. This map was created in ArcGIS desktop 10.7.

The study area is located in Lin’an city (29°–31° N, 118°–120° E), Zhejiang province, southeastern China (Fig. 5). It is the largest production area of Chinese hickory, accounting for approximately 51% of the nationally planted areas^79^. The Chinese hickory planting densities range from 300 to 375 stems ha^−1^, with an average diameter at breast height (DBH) of 12 cm and an average tree height of 8 m^80^. The area is characterized by subtropical monsoon climate with four distinct seasons, with the annual average temperature of 16 °C and annual precipitation of 1628 mm. The annual average daylight hours are 1774 h with 235 d frost-free^81^. It has undulating topography with an elevation range of 150–1000 m^82^. The forest ages range of hickory plantations from 30–100 years. According to Geology online map of Zhejiang Province, China (https://www.osgeo.cn/map/m02cd) and Dong et al.^83^, the soil is derived from 7 major types of parent material, which include sandstone, sand shale, slate, phyllite, rhyolite, granite and quartz porphyry. During the period of 2008–2010, 600–800 kg ha^−1^ of a compound fertilizer (N:P_2_O_5_:K_2_O, 15:15:15) was applied every year^41^. And herbicide application and mowing were the main methods for controlling understory vegetation. In order to reduce agricultural non-point source pollution, reduce soil erosion and phosphorus pollution, improve soil fertility, the local government issued a document, the application of compound fertilizer was reduced to 150–300 kg ha^−1^ per year, herbicide was banned, and grass was planted under the forest after 2010.

### Field sampling and laboratory analysis

According to the f distribution characteristics of China hickory plantations of Lin 'an district, a grid of 1 km × 1 km were set up. And the grids containing the hickory plantations was taken as sample plots. A total of 209 sample sites were established in 2008. For each site, a 20 m × 20 m plot was set in the center of each sample plot. Five subsoil samples with a depth of 0–30 cm were collected at 2–2.2 m from the tree trunk (the main distribution area of hickory root system) according to the "Z" shape, which were further mixed into one soil sample with a weight of 1 kg, and brought back to laboratory. For each sampling year, 209 soil samples were collected in situ in July of 2008, 2013 and 2018, respectively (Fig. 5). A portable global positioning system (GPS) was used to record the coordinates and altitude of each sampling location. Information on parent materials and forest age were recorded in 2008. The survey related to management measures (including fertilization, weeding and harvesting methods) of the Chinese hickory plantation regions was also carried out every 5 years. Mean average precipitation (MAP) and mean average temperature (MAT) information of sample plots come from the weather forecast network.

After all soil samples were air-dried, the visible root debris was removed and sieved through 2-mm nylon mesh. A portion of each soil sample was ground with an agate mortar to pass the 0.149 mm nylon mesh, and sealed in an enclosed polyethylene bag. The air-dried samples were prepared for physicochemical analysis. All experimental methods were referred to Lu^59^. **S**oil pH was determined using a pH meter at soil/water (*w/v*) ratio of 1:2.5. The soil AN was measured by a diffusion method. Soil AK was extracted with 1 mol L^−1^ NH_4_Ac, and then measured by an atomic absorption spectrometer. The AP was extracted using 0.5 mol L^−1^ NaHCO_3_ (pH = 8.5) and the P concentration in the extract was determined using the molybdenum-blue method. SOC was determined by the K_2_Cr_2_O_7_–H_2_SO_4_ digestion, and titration with ammonium sulfate iron (Fe (NH_4_)_2_(SO_4_)_2_·6H_2_O) solution.

### Data analysis

#### Descriptive statistics and difference tests

The maximum, minimum, range, mean, standard deviation (SD), CV, kurtosis, skewness and significance test of sample indexes for 2008, 2013 and 2018 were presented. Test of normality for soil pH, AN, AP, AK, SOC was performed by the Kolmogorov–Smirnov (K-S) test^84^. None of the data were normally distributed. Therefore, the Box-Cox transformation of soil properties were performed to meet the assumption of normality using Matlab r2019a software. Kernel density estimation was used to estimate the distribution of the soil properties in all sample plots (package stats in R statistical software 4.0.0). One-way ANOVA was used to compare the differences in soil properties in 2008, 2013 and 2018. Pearson correlation analysis was used to identify the correlations between soil pH, AN, AP, AK and SOC^47^. An alpha level of 0.05 for significance testing was used in all statistical analyses, unless mentioned otherwise.

#### Spatial autocorrelation analyses

Spatial autocorrelation analysis is a statistical method to measure the cluster degree of spatial variables^85^. Moran's *I* is a commonly used index of spatial autocorrelation, which reflects the similarity between adjacent samples^86^. The global Moran’s *I* was used to describe the soil properties autocorrelation feature over the entire regions (see Supplementary material, Text S1 for detailed information).

#### Geostatistical analysis

The semi-variance (or variogram) is widely used in geostatistics to quantitatively describe the spatial variability of environmental variables, and this relationship was expressed through an effective variogram model, which can further provide input parameters for spatial interpolation of kriging^87^ (see Supplementary material, Text S2 for detailed information).

The ordinary Kriging method can be used to derive the optimal linear unbiased estimate of spatial variables^88^. The models that fit the semivariogram best according to the regression coefficient were determined. For the kriging interpolation, the transformed soil properties data were used. The ordinary kriging method was used to draw a spatial distribution map of soil properties and soil fertility index with ArcGIS desktop 10.7 (https://www.esri.com/en-us/arcgis/products/arcgis-desktop/resources).

#### Soil fertility evaluation

The calculation of Integrated Fertility Index (IFI) comprises three steps: (1) the selection of indicators, (2) the calculation of the individual fertility index (IFI_*i*_), and (3) the calculation of IFI. The soil pH, AN, AP, AK and SOC were used in the calculations. To calculate the IFI the following equation was used:

1 $$ {\text{IFI}}_{i}  = {\text{ }}\left\{ {\begin{array}{*{20}l}    {\frac{x}{{x_{a} }}} \hfill & {x{\text{ <  }}x_{a} } \hfill  \\    {1 + \frac{{(x - x_{a} )}}{{(x_{b}  - x_{a} )}}} \hfill & {x_{a}  \le x \le x_{b} } \hfill  \\    {2 + \frac{{(x - x_{b} )}}{{(x_{c}  - x_{b} )}}} \hfill & {x_{a}  \le x \le x_{b} } \hfill  \\    3 \hfill & {x{\text{  > }}x_{c} } \hfill  \\   \end{array} } \right. $$

where IFI_*i*_ is the individual fertility index; *x* is the measured value of each attribute^89^; *x*_*a*_* x*_*b*_ and *x*_*c*_ are the upper and lower limits of each classification standard based on forest soils in Zhejiang. In the process of carrying out the cultivated land soil survey and evaluation project, soil scientists developed the soil property classification standard of Zhejiang Province. Soil properties such as pH, SOC, AP and AK are the main controlling factors of soil fertility, while AN is not considered to be the main controlling factor^59^. And according to the requirements of nut species on soil acidity, soil nutrient. The classification criteria and separate weights were determined as shown in Table S1^90^.

The final step was to calculate IFI using the improved Nemerow Quality Index equation:

2 $$IFI = {\sqrt {\frac{1}{2}\left(IFI_{i\text{ave}}^{2} + IFI_{i\text{min} }^{2}\right)}} \times \left( {\frac{n - 1}{n}} \right)$$

where IFI is the soil integrated fertility index; IFI_*i*ave_ is the average values for the individual fertility indices; IFI_*i*min_ is the minimum value for the individual fertility indices; *n* is the number of soil properties^91^. The degree of IFI was classified as follows: IFI < 0.9; low, 0.9 ≤ IFI < 1.8; moderate, 1.8 ≤ IFI < 2.7; high, and IFI ≥ 2.7; very high.

## Supplementary Information

Supplementary Information.
