# Supplementary material for: A 10-year monitoring of soil properties dynamics and soil fertility evaluation in Chinese hickory plantation regions of southeastern China
Source: Sci Rep. 2021 Dec 7;11:23531. doi: 10.1038/s41598-021-02947-z (PMC8651749; doi:10.1038/s41598-021-02947-z)
Supplement: Supplementary file 1 — Supplementary Information. [file 41598_2021_2947_MOESM1_ESM.docx]

**A 10-year monitoring of soil properties dynamics and soil fertility evaluation in Chinese hickory plantation regions of southeastern China**

**Jin Jin^a,1^, Luoqi Wang^a,1^, Karin Müller^b^, Jiasen Wu^a,*^, Hailong Wang^c^, Keli Zhao^a^, Frank Berninger^d^, Weijun Fu^a,e,*^**

^a^ State Key Laboratory of Subtropical Silviculture, Zhejiang A&F University, Hangzhou, 311300, China

^b^ The New Zealand Institute for Plant & Food Research Limited, Ruakura Research Centre, Private Bag, Hamilton, 3123, New Zealand

^c^ Biochar Engineering Technology Research Center of Guangdong Province, School of Environmental and Chemical Engineering, Foshan University, Foshan, 528000, China

^d^ Department of Environmental and Biological Sciences, University of Eastern Finland, PO Box 111, Joensuu, 80101, Finland

^e^ Zhejiang Provincial Key Laboratory of Carbon Cycling in Forest Ecosystems and Carbon Sequestration, Zhejiang A&F University, Lin’an 311300, China

### Supplementary material

**Text S1. The detailed information of Moran’s I**

global Moran's *I* (I) can be expressed as:

$I=\left[\frac{N}{\sum_{i=1}^{N} \sum_{j=1}^{N} w_{ij}}\right]\left[\frac{\sum_{i=1}^{N} \sum_{J=1}^{N} \text{Ω}_{ij}(Z_{i}-\overline{Z})(Z_{j}-\overline{Z})}{\sum_{i=1}^{N} (Z_{i}-\overline{Z})^{2}}\right]$ equation (1)

where *N* is the number of points, with *i* and *j* taking values from 1 to N, assuming that there are n spatial samples.‾*Z* is the mean value of *Z*; *Z_i_* and *Z_j_* are the values of variable at locations *i* and *j*, respectively (*i* ≠ *j*); and w*_ij_* is the spatial weight describing the adjacency of distance between the *i*^th^ and *j*^th^ point^1,2^. A single value is used to reflect the degree of autocorrelation of variables. When calculating the global Moran's *I* statistics, two parameters, Z(I) and *P-*value provide the calculated Moran’s I statistics statistical significance^3^. The Z(I) is calculated as follows:

$\text{Z(I)}=\text{(I}-\text{E(I)/}\sqrt{\text{Var(I)}})$ equation (2)

where I represents Moran’s I, and E(I) and Var(I) are the theoretical expectation and variance of Moran’s I, respectively.

While global Moran’s *I* represent the presence or absence of spatial autocorrelation as a whole, Anselin (1995)^3^ defines such local correlation statistics as local indicators of spatial association (LISA), which can be used to detect spatial clustering or "hot spots". The local Moran’s *I* is one of LISA, represents the significant spatial clustering of similar values around a particular observation^4,5^. The statistic for Local Moran’s *I* is defined as:

$\text{I}_{i}=\frac{\text{Z}_{i}-\overline{\text{Z}}}{\text{σ}^{2}}\sum_{j=1j\neq i}^{N} \left[w_{ij}(Z_{j}-\overline{Z}\text{）}\right]$ equation (3)

Where‾*Z* is the mean value of *Z* with the sample number of n; *Z_i_* is the value of the variable at location *i*; Z*_j_* is the value at other locations (where *j*≠*i*); σ^2^ is the variance of *Z*; and W*_ij_* is a distance weighting between *Z_i_* and *Z_j_*.

Local Moran's *I* can detect spatial outliers, locates spatial associations, and identifies local clusters^1,5^. The local Moran's *I* index was applied to identify the soil properties cluster in this study. Global Moran’s *I* and local Moran’s *I* were calculated using the GeoDa software (Version 1.14).

**Text S2. The detailed information of Semi-variance**

The standard equation for the semivariance is given as [Eq. (4)]:

$\text{γ}(h\text{) }=\frac{1}{\text{2N(}h\text{)}}\sum_{i=1}^{N(h)} \left[Z(x_{j})-Z(x_{i}+h)\right]^{2}$ equation (4)

where γ (*h*) is the experimental semivariance value at distance interval *h* (km) and describes the degree of autocorrelation, *Z*(*x_i_*) and *Z*(*x_i_+h*) are the measured sample value at grid *x_i_* and sample value at *x_i_+h*, respectively, and N(*h*) is the total number of sample data pairs within distance *h* (km). The following parameters are applied to identify the semivariance functions^6-9^: sill (C_0_+C), range (A_0_), nugget effect (C_0_), spatially dependent structural variance (C), and the ratio of the Nugget effect (C_0_) to the Sill (C_0_+C). The "Nugget to-sill" ratio ([C_0_/(C_0_+C)]) is an important indicator to measure the extent of spatial dependence of variables in soil^10^. The ratio < 0.25 indicates that the studied variable has a strong spatial dependencies, which is mainly controlled by intrinsic variation, such as topography, soil type, parent material, climate. The ratio between 0.25 and 0.75 shows moderate spatial dependence of the studied variable. The ratio > 0.75 reveals that the study variable has weak spatial dependence, and is mainly affected by external factors (human activities, such as mining, road traffic, fertilization, tillage, planting system, land use intensity, etc.). The experimental semivariance was fitted by exponential model, Gaussian model, spherical model and linear model. Analysis of semivariance was carried out with GS+ (Version 7.0).


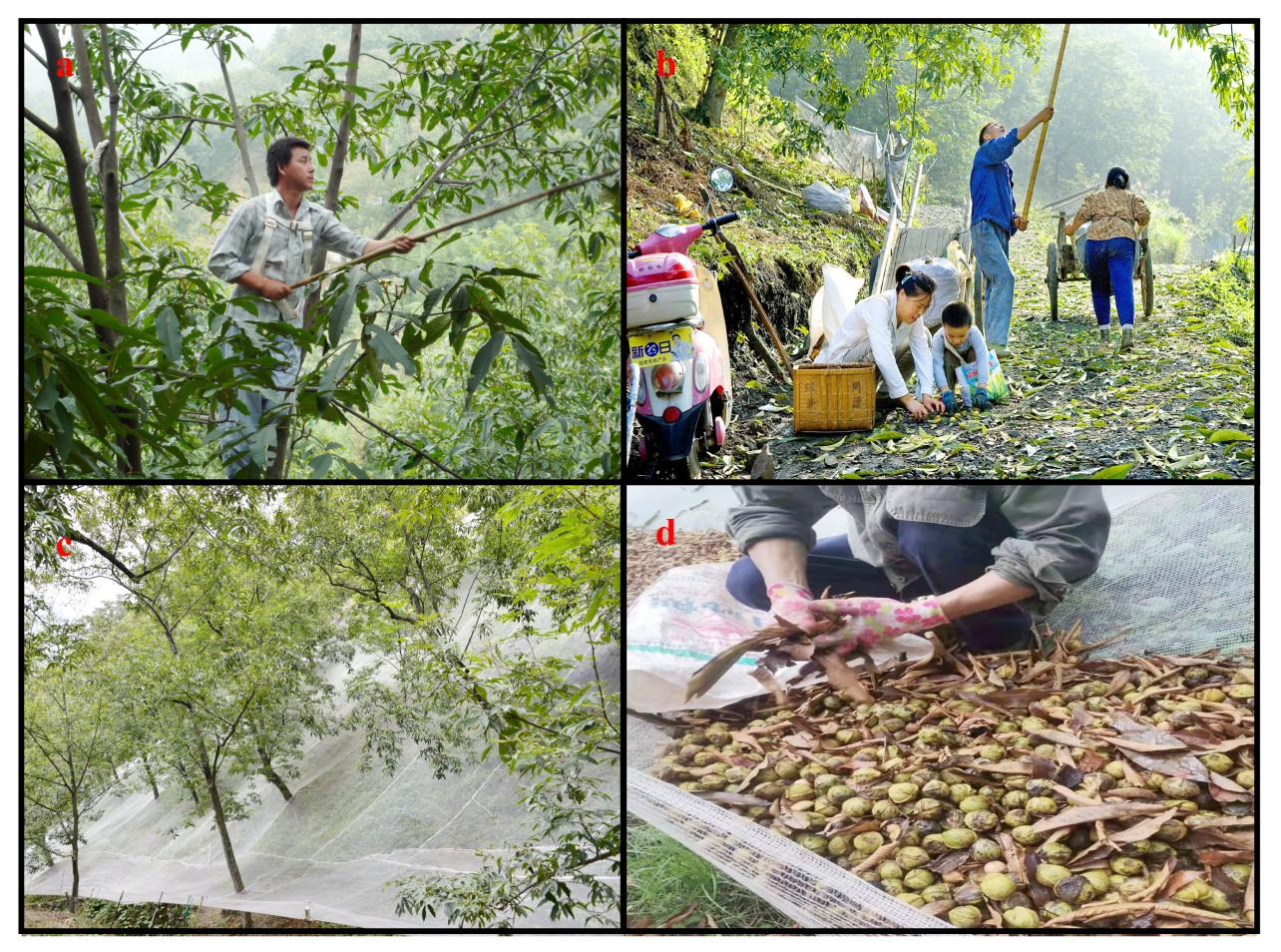


**Figure S1.** Comparison of knock and net harvesting. a and b represent knock harvesting, c and d represent net harvesting.


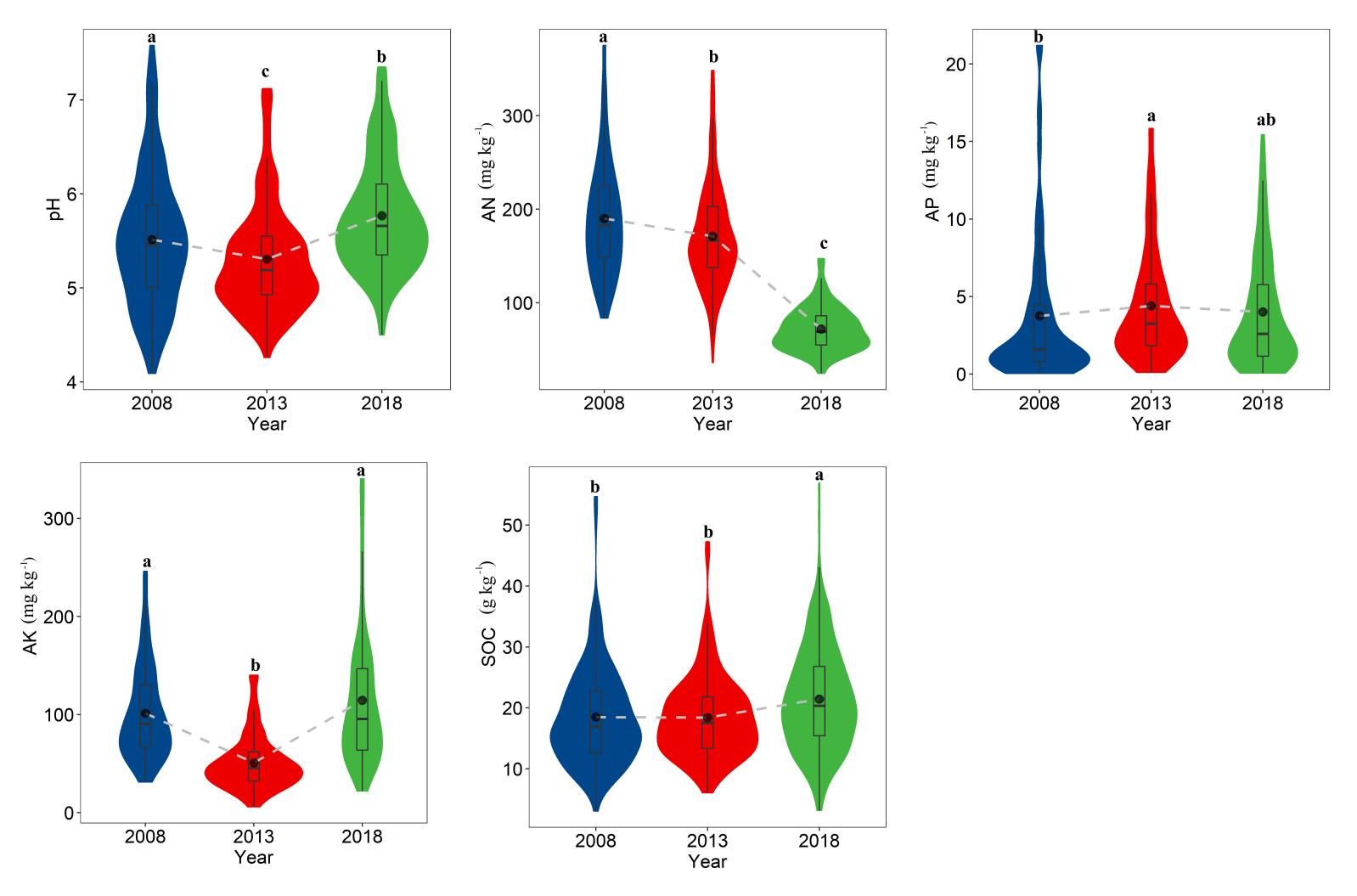


**Figure S2.** Kernel density estimation of soil properties in 2008, 2013 and 2018. a, b and c indicate significant differences among years at *P*< 0.05. level. Black points represent the mean value and black box represent the distributions of quarter value, median value and three-quarters value. AN: available nitrogen; AP: available phosphorus; AK: available potassium; SOC: soil organic carbon.

**
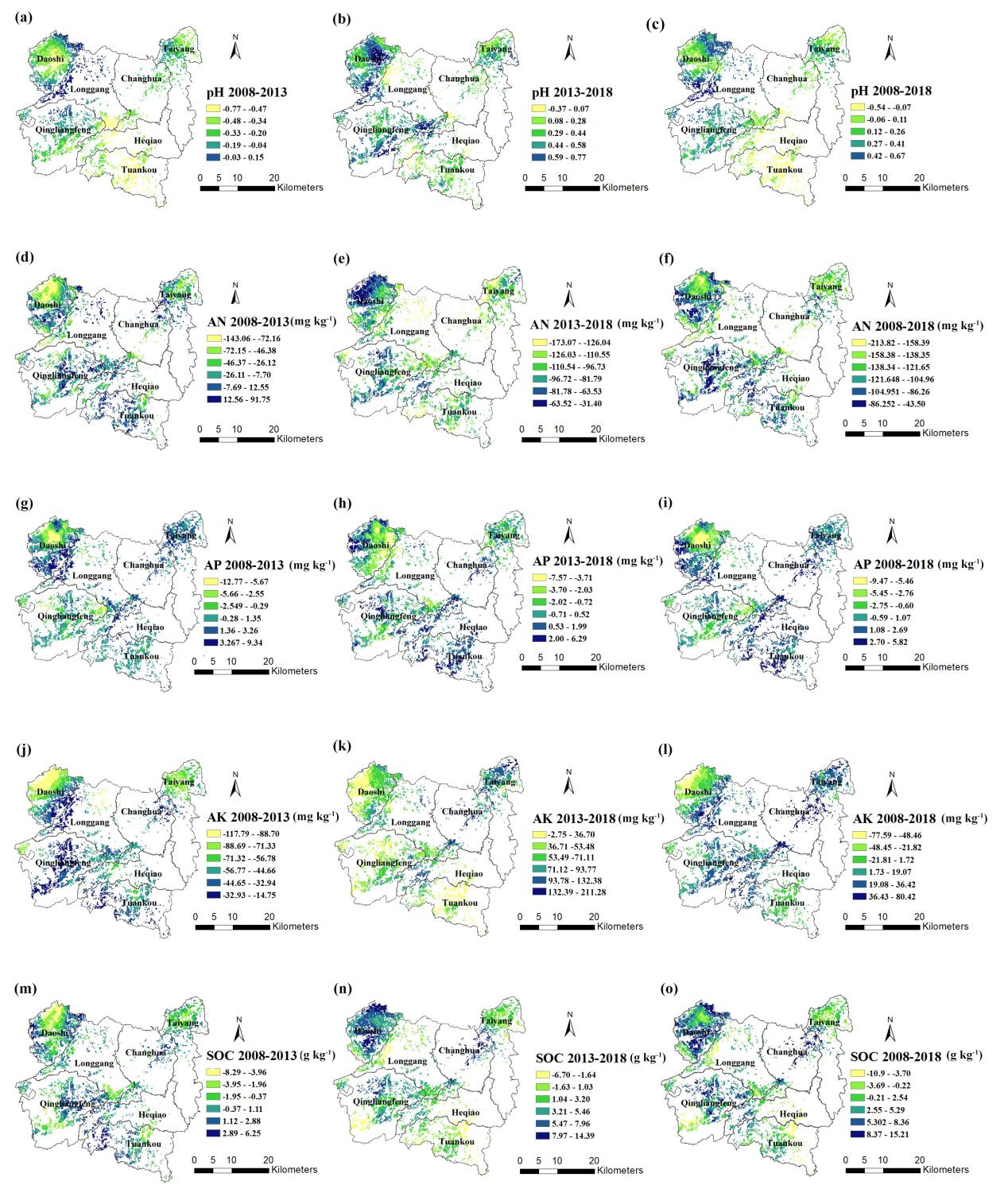
**

**Figure S3.** Spatial variation of the change of soil properties in hickory plantation regions. AN: available nitrogen; AP: available phosphorus; AK: available potassium; SOC: soil organic carbon. The maps were created in ArcGIS desktop 10.7.


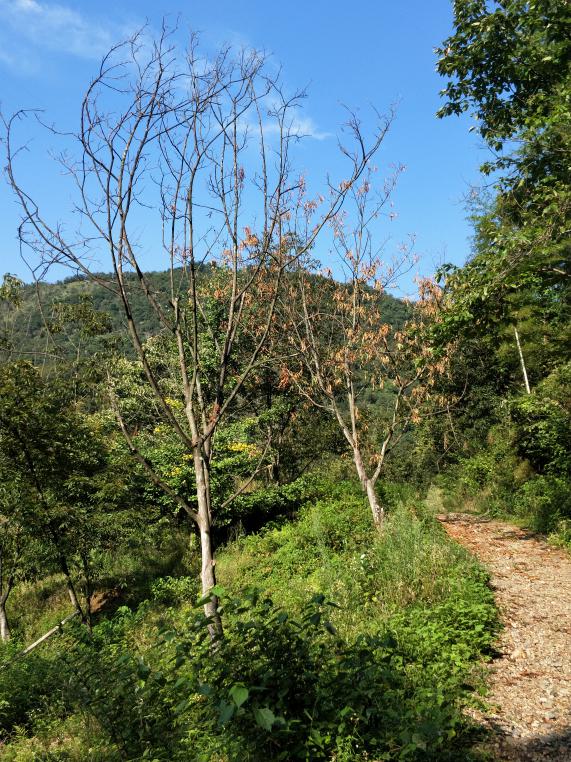


**Figure S4.** Overuse of herbicides causes death of hickory

**Table S1.** Thresholds values and weights for the quantitative soil quality indicators.

|  | *x_a_* | *x_b_* | *x_c_* | Weights |
| --- | --- | --- | --- | --- |
| pH | 4.50 | 5.50 | 6.50 | 0.20 |
| AN (mg kg^-1^) | 60.00 | 120.00 | 180.00 | 0.10 |
| AP (mg kg^-1^) | 5.00 | 10.00 | 20.00 | 0.15 |
| AK (mg kg^-1^) | 50.00 | 100.00 | 200.00 | 0.25 |
| SOC (g kg^-1^) | 5.80 | 11.60 | 17.40 | 0.30 |

*x_a_, x_b_* and *x_c_* are the low, medium and high limits of each classification standard, respectively. AN: available nitrogen; AP: available phosphorus; AK: available potassium; SOC: soil organic carbon.

**Table S2.** Main effect factors of the change in soil properties.

| Attributes | Factors | Type Ⅲsum of squares | df | Mean square | The F statistic | F value with probability |
| --- | --- | --- | --- | --- | --- | --- |
|  |  |  |  |  |  |  |
| C_pH_ | Altitude | 2.552 | 3 | 0.851 | 2.504 | *** |
|  | MAP | 7.684 | 2 | 3.842 | 7.667 | ** |
|  | MAT | 13.507 | 4 | 4.502 | 9.935 | *** |
|  | Parent material | 2.607 | 6 | 0.434 | 1.271 | *** |
|  | Forest age | 1.576 | 3 | 0.525 | 1.005 | *** |
|  | Fertilizer | 3.482 | 2 | 1.741 | 5.261 | ** |
|  | Harvest method | 5.621 | 4 | 1.405 | 4.343 | 0.223 |
|  | Weeding method | 4.813 | 2 | 2.406 | 7.419 | *** |
| C_AN_（mg kg^-1^） | Altitude | 9150.211 | 3 | 3050.070 | 5.922 | 0.097 |
|  | MAP | 14417.277 | 2 | 7208.639 | 1.983 | 0.167 |
|  | MAT | 31026.333 | 4 | 10342.111 | 2.94 | 0.069 |
|  | Parent material | 4548.592 | 6 | 758.099 | 1.402 | 0.216 |
|  | Forest age | 18798.416 | 3 | 6266.139 | 1.776 | * |
|  | Fertilizer | 7313.444 | 4 | 1828.361 | 3.504 | * |
|  | Harvest method | 6121.085 | 1 | 6121.085 | 11.771 | 0.701 |
|  | Weeding method | 3301.337 | 2 | 1650.668 | 3.077 | * |
| C_AP_（mg kg^-1^） | Altitude | 154.680 | 3 | 51.560 | 4.003 | 0.103 |
|  | MAP | 87.538 | 2 | 43.769 | 1.431 | 0.012 |
|  | MAT | 251.901 | 4 | 83.967 | 2.857 | ** |
|  | Parent material | 149.741 | 6 | 24.957 | 1.922 | 0.629 |
|  | Forest age | 78.551 | 3 | 26.184 | 0.905 | * |
|  | Fertilizer | 158.985 | 4 | 39.746 | 3.103 | * |
|  | Harvest method | 131.917 | 1 | 131.917 | 10.344 | ** |
|  | Weeding method | 192.347 | 2 | 96.174 | 7.682 | 0.695 |
| C_AK_（mg kg^-1^） | Altitude | 29083.165 | 3 | 9694.388 | 2.012 | ** |
|  | MAP | 19263.247 | 2 | 9631.623 | 1.484 | 0.105 |
|  | MAT | 34908.457 | 4 | 11636.152 | 1.816 | 0.117 |
|  | Parent material | 29276.957 | 6 | 4879.493 | 1.006 | 0.570 |
|  | Forest age | 14823.104 | 3 | 4941.035 | 0.709 | 0.153 |
|  | Fertilizer | 50958.698 | 4 | 12739.675 | 2.713 | * |
|  | Harvest method | 14803.068 | 1 | 14803.068 | 3.082 | ** |
|  | Weeding method | 18606.03 | 2 | 9303.015 | 1.935 | 0.936 |
| C_SOC_（g kg^-1^） | Altitude | 1091.888 | 3 | 363.963 | 5.650 | *** |
|  | MAP | 237.919 | 2 | 118.959 | 1.329 | 0.119 |
|  | MAT | 347.224 | 4 | 115.741 | 1.296 | 0.070 |
|  | Parent material | 467.790 | 6 | 77.965 | 1.138 | 0.280 |
|  | Forest age | 188.251 | 3 | 62.750 | 0.631 | * |
|  | Fertilizer | 1332.535 | 4 | 333.134 | 5.241 | ** |
|  | Harvest method | 517.803 | 1 | 517.803 | 7.774 | 0.077 |
|  | Weeding method | 985.365 | 2 | 492.682 | 7.622 | 0.578 |

* *P*<0.05, ** *P*<0.01, *** *P*<0.001. MAP: Mean annual precipitation; MAT: Mean annual temperature; C_pH_: change in pH; C_AN_: change in available nitrogen; C_AP_: change in available phosphorus; C_AK_: change in available potassium; and C_SOC_: change in soil organic carbon.

**Reference**

1 Fu, W. *et al*. Outlier identification of soil phosphorus and its implication for spatial structure modeling. *Precis. Agric.* **17**, 121-135; 10.1007/s11119-015-9411-z (2016).

2 Shi, A. *et al.* Long-term effect of E-waste dismantling activities on the heavy metals pollution in paddy soils of southeastern China. *Sc. Total Environ.* **705**, 135971; 10.1016/j.scitotenv.2019.135971 (2020).

3 Anselin, L. *et al.* Local Indicators of Spatial Association-LISA. *Geogr. Anal.* **27**, 93-115; 10.1111/j.1538-4632.1995.tb00338.x (1995).

4 Zhao, K. *et al.* Spatial variations of concentrations of copper and its speciation in the soil-rice system in Wenling of southeastern China. *Environ. Sci. Pollut. R.* **21**, 7165-7176; 10.1007/s11356-014-2638-9 (2014).

5 Fu, W. *et al.* Spatial variation of biomass carbon density in a subtropical region of Southeastern China. *Forests* **6**, 1966-1981; 10.3390/f6061966 (2015).

6 Treitz, P. & Howarth, P. High spatial resolution remote sensing data for forest ecosystem classification: An examination of spatial scale. *Remote Sens. Environ.* **72**, 268-289; 10.1016/S0034-4257(99)00098-X (2000).

7 Zawadzki, J. *et al.* Applying geostatistics for investigations of forest ecosystems using remote sensing imagery. *Silva Fenn.* **39**, 599-618; 10.14214/sf.369 (2005).

8 Fu, W. *et al.* Using Moran's *I* and GIS to study the spatial pattern of forest litter carbon density in a subtropical region of southeastern China. *Biogeosciences* **11**, 2401-2409; 10.5194/bg-11-2401-2014 (2014).

9 Chen, S. *et al.* Spatial and temporal changes of soil properties and soil fertility evaluation in a large grain-production area of subtropical plain, China. *Geoderma* **357**,113937 ;10.1016/j.geoderma.2019.113937 (2020).

10 Cambardella, C. A. *et al.* Field-scale variability of soil properties in central Iowa soils. *Soil Sci. Soc. Am. J.* **58**, 1501-1511; 10.2136/sssaj1994.03615995005800050033x (1994).
